# Severe Uncompensated Metabolic Alkalosis due to Plasma Exchange in a Patient with Pulmonary-Renal Syndrome: A Clinician's Challenge

**DOI:** 10.1155/2015/802186

**Published:** 2015-06-08

**Authors:** Mohsin Ijaz, Naeem Abbas, Dmitry Lvovsky

**Affiliations:** ^1^Division of Pulmonary and Critical Care Medicine, Department of Medicine, Bronx Lebanon Hospital Center, 1650 Selwyn Avenue, Suite 12F, Bronx, NY 10457, USA; ^2^Department of Medicine, Bronx Lebanon Hospital Center, 1650 Selwyn Avenue, Suite 10C, Bronx, NY 10457, USA

## Abstract

Metabolic alkalosis secondary to citrate toxicity from plasma exchange is very uncommon in patients with normal renal function. In patients with advanced renal disease this can be a fatal event. We describe a case of middle-aged woman with Goodpasture's syndrome treated with plasma exchange who developed severe metabolic alkalosis. High citrate load in plasma exchange fluid is the underlying etiology. Citrate metabolism generates bicarbonate and once its level exceeds the excretory capacity of kidneys, the severe metabolic alkalosis ensues. Our patient presented with generalized weakness, fever, and oliguria and developed rapidly progressive renal failure. Patient had positive serology for antineutrophilic cytoplasmic antibodies myeloperoxidase (ANCA-MPO) and anti-glomerular basement membrane antibodies (anti-GBM). Renal biopsy showed diffuse necrotizing and crescentic glomerulonephritis with linear glomerular basement membrane staining. Patient did not respond to intravenous steroids. Plasma exchange was started with fresh frozen plasma but patient developed severe metabolic alkalosis. This metabolic alkalosis normalized with cessation of plasma exchange and initiation of low bicarbonate hemodialysis. ANCA-MPO and anti-GBM antibodies levels normalized within 2 weeks and remained undetectable at 3 months. Patient still required maintenance hemodialysis.

## 1. Introduction

Anti-glomerular basement membrane antibody disease is a rare but well-recognized cause of glomerulonephritis. The incidence is reported to be one case per one million population [1]. About 60–70% of the affected patients present with pulmonary involvement in the form of alveolar hemorrhage [2]. In the setting of advanced renal failure, metabolic alkalosis (MA) is an uncommon phenomenon. Citrate is used as an anticoagulant for plasma exchange fluid and its in vivo conversion into bicarbonate leads to the metabolic alkalosis and its attendant complications. Double positive (serum positive for anti-GBM and ANCA-MPO) Goodpasture's disease is associated with worse renal outcomes and tobacco smoking increases chances of relapse of disease [2]. Aggressive treatment strategies in the form of immunosuppressive medications and plasmapheresis are the mainstay of treatment [3]. Failure to respond to conservative management can lead to the need for hemodialysis (HD).

## 2. Case Presentation

A 54-year-old woman presented with generalized body aches, weakness, back pain, and fever of one-week duration. She had medical history of hypertension, depression, osteoarthritis, and smoking. Patient felt generalized weakness and had decrease in urine output, with dysuria and dark colored urine.

On admission, she was hemodynamically stable, alert, coherent, and oriented. Cardiovascular examination showed normal heart sounds, with no murmur, rub, or gallops. Respiratory examination revealed equal bilateral air movements with no adventitious sounds. Abdomen was soft, nontender with no organomegaly. Neurological examination showed intact cranial nerves with no motor or sensory deficit. There was no leg edema or cutaneous manifestation of vasculitis.

**Figure 1:**
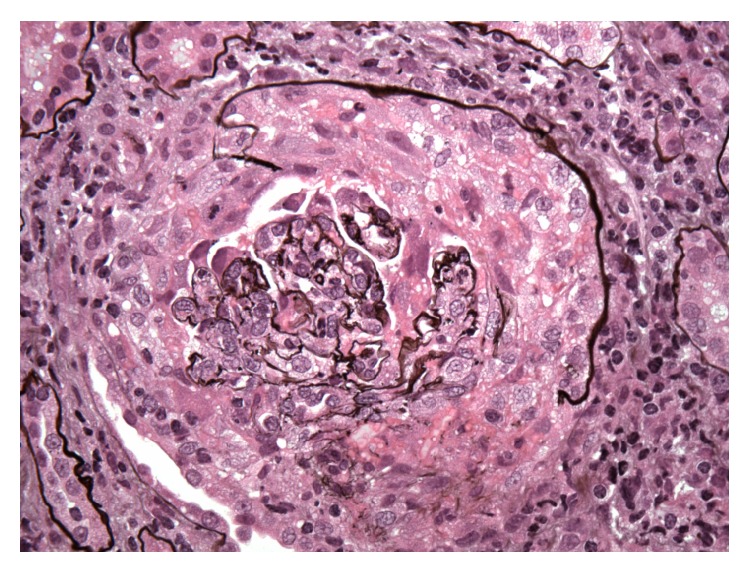
Diffuse necrotizing and crescentic glomerulonephritis.

**Figure 2:**
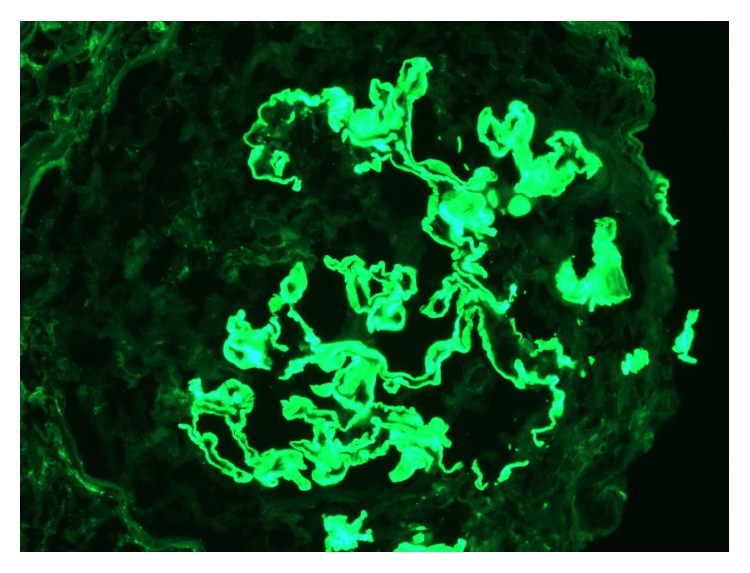
Immunofluorescence stain with linear glomerular basement membrane staining for IgG.

**Figure 3:**
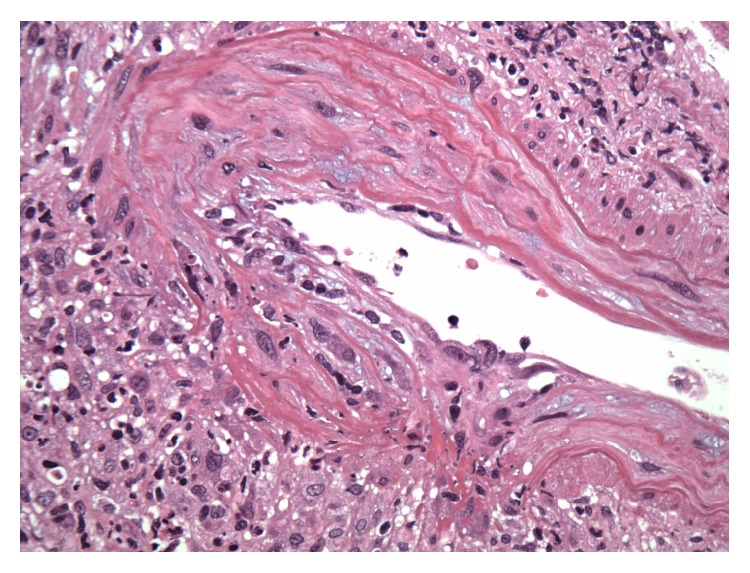
Medium caliber vessel shows transmural arteritis with disruption of the elastic and focal fibrinoid necrosis.

**Figure 4:**
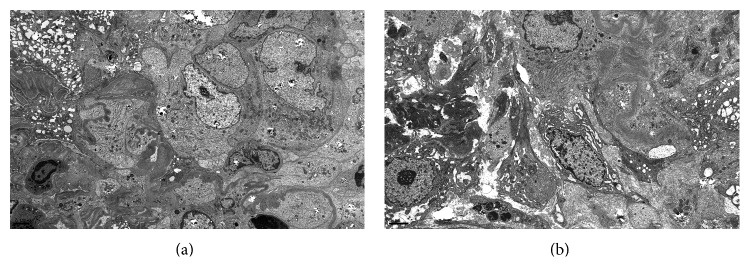
Electron microscopy shows all of the glomeruli sampled revealing global involvement by cellular crescents. Focal areas of GBM rupture associated with fibrin extravasation are noted. No immune deposits are identified. Tubules show degenerative changes and the interstitium contains patchy moderate inflammation.

Patient was noted to be in rapidly progressing acute renal failure and anemia. Autoimmune work-up revealed positive ANCA-MPO and anti-GBM antibody. Renal sonogram showed normal sized kidneys and no signs of obstruction. Renal biopsy showed diffuse necrotizing and crescentic glomerulonephritis (GN) with linear GBM staining consistent with acute, severe anti-GBM nephritis; ANCA associated focal necrotizing vasculitis; moderate tubular atrophy; interstitial fibrosis (Figures 1, 2, 3, and 4). She was given pulse intravenous methylprednisolone therapy (1000 mg daily) for three days and later on switched to tapering doses of oral prednisone. Patient was given trial of cyclophosphamide which was abandoned because of intolerance due to recalcitrant nausea and vomiting and gross hematuria.

**Figure 5:**
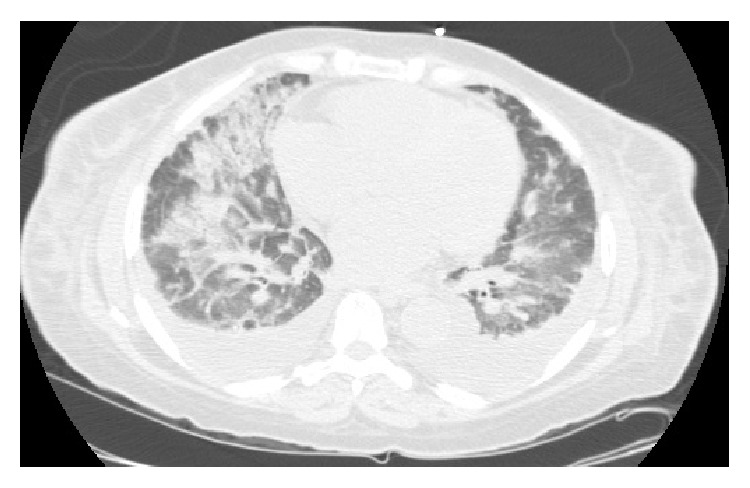
CT chest showing bilateral alveolar infiltrates and ground glass opacities.

**Figure 6:**
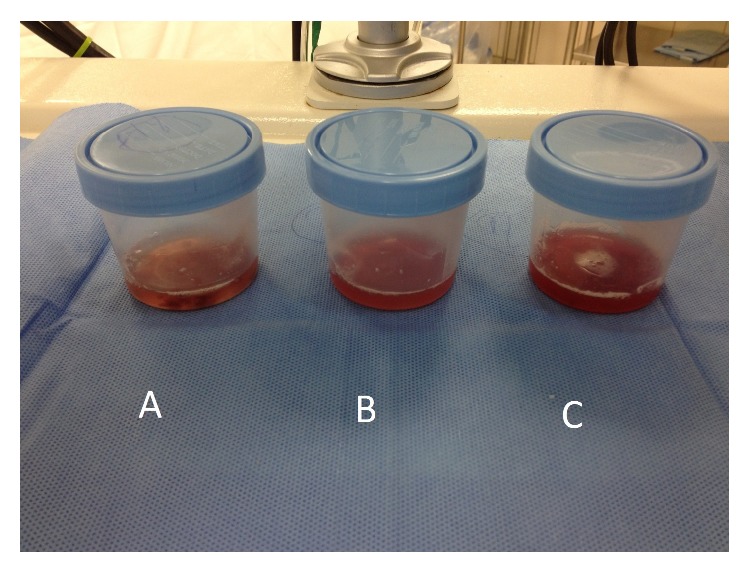
Bronchoalveolar fluid showed evidence of diffuse alveolar hemorrhage (A–C).

In the next few days, she developed sudden shortness of breath, cough with hemoptysis, and hypoxia with oxygen saturation dropping down to 84% on ambient air, with crackles at lung bases bilaterally. She had acute hypoxic respiratory failure requiring orotracheal intubation and mechanical ventilation with full sedation. Computerized tomography (CT) of the chest revealed ground glass opacities and interstitial changes bilaterally (Figure 5). Given the acute onset of hypoxia, with no obvious source and explanation, bronchoscopy was performed which showed diffuse alveolar hemorrhage (Figure 6). Bronchoalveolar lavage was negative for any bacterial growth.

The patient was started on plasma exchange with fresh frozen plasma and hemodialysis for nonresolving acute renal failure. During the process of plasmapheresis with fresh frozen plasma, her laboratory data were noted to be significant for severe MA and respiratory alkalosis with pH ranging up to 7.67, HCO_3_^−^ level going up to 34, and low ionized calcium of 0.94. Metabolic component of alkalosis was predominant from the high citrate load given during plasmapheresis (Table 1). All these parameters were reversed with cessation of plasmapheresis. The patient was continued on low bicarbonate HD. There was appropriate response in the level of anti-GBM and ANCA-MPO level, but no sustained improvement in renal function was noted and patient ended up in receiving long term HD.

## 3. Discussion

Goodpasture's syndrome is a rare disorder of acute glomerulonephritis and alveolar hemorrhage due to autoantibodies binding to the alveolar and glomerular basement membranes. It affects mainly young individuals, men being more commonly affected, and the majority of them are smokers [3]. Slightly fewer than half of them can present with pulmonary symptoms such as shortness of breath, hypoxia, and hemoptysis. Alveolar hemorrhage can be readily and reliably diagnosed by bronchoalveolar lavage. The renal outcome heavily depends on the initial serum creatinine and end-stage renal disease requiring hemodialysis or renal transplantation occurs in up to 42% of the individuals [2].

Serum anti-GBM antibody is a very specific marker of the disease and its level is correlated with the severity of the disorder. About 10%–40% of Goodpasture's syndrome patients will also have serum ANCA antibodies. The recognition of ANCA is very important as this group of patients is more likely to have treatable disease than others who have only anti-GBM antibodies [2]. Plasma exchange remains the modality of choice. The other options are corticosteroids and other immunosuppressive agents such as cyclophosphamide [4].

MA is frequently encountered in the inpatient setting. It may result in severe tissue hypoxia from compensatory alveolar hypoventilation and left shift of the oxyhemoglobin curve. The other dreaded complication is cardiac arrhythmias from alkalemia, associated low serum ionized calcium, and hypokalemia [5]. Severe MA with pH more than 7.55 is an emergent condition. In one study, Wilson et al. showed that a rising pH was associated with increased mortality (41% with pH 7.55 to 7.56, 47% with pH 7.57 to 7.59, 65% with pH 7.6 to 7.64, and 80% with pH 7.65 to 7.7) [6].

Plasma exchange is a well-established tool for the management of various immune and nonimmune conditions. Plasmapheresis is thought to remove the large molecular weight antibodies and proinflammatory markers. The development of MA in the context of plasma exchange has been described in many conditions such as systemic lupus erythematous, Goodpasture's syndrome, and ANCA vasculitis, suggesting that the underlying pathology is with plasma exchange rather than the underlying medical condition for which plasmapheresis is being used [7–9]. Three molecules of bicarbonate are generated from one molecule of citrate. Our patient was anuric and so unable to eliminate excess bicarbonate generated from citrate. Modifications in the plasma exchange protocol like albumin solution or cryoprecipitate with no citrate load, rather than fresh frozen plasma (FFP) which requires citrate for anticoagulation purpose, can prevent this complication. Citrate induced MA in individuals with renal insufficiency can be managed by HD following therapeutic plasma exchange. The overall mortality reported with plasmapheresis is 0.03–0.05 [10]. The most common complications reported are cardiac and respiratory [10].

There are two main mechanisms for the development of MA in the setting of advanced renal disease. Exogenous mechanism occurs in the form of ingestion or treatment with base therapy. Endogenous pathway acts in the form of HCO_3_^−^ reclamation in the tubules in the setting of hypovolemia or loss of Na^+^, Cl^−^, or H^+^, usually due to diuretics therapy or vomiting [11]. FFP is anticoagulated with sodium citrate at 14 g/dL. The metabolism of sodium citrate (C_6_H_5_O_7_Na_3_) to CO_2_ and H_2_O finally yields 3 molecules of NaHCO_3_ [12]. The renal tubules have the extraordinary capacity to absorb the citrate and metabolic alkalosis slows down the net reabsorption of citrate. All the excreted citrate metabolizes to CO_2_ which is about 15% of total renal production of CO_2_ [13].

Pearl and Rosenthal [9] described that MA can be prevented if 3 percent of albumin or cryoprecipitate rather than FFP is used as replacement for the removal of patient plasma. Hsu et al. [5] used normal bicarbonate hemodialysate of 25 to 28 mEq/L to successfully treat the severe MA secondary to plasma exchange. Plasmapheresis has been recommended for the treatment of anti-GBM disease, with the aim of removing circulating pathogenic antibodies. It results in rapid reduction of the anti-GBM levels as observed in our case [1]. In 2007, the American Society for Apheresis guidelines recommend that all patients with anti-GBM antibody, not already on dialysis, should undergo intensive plasma exchange for at least 14 days or until anti-GBM antibodies become undetectable [14].

Our patient had similar clinical course. She developed severe MA with attendant biochemical parameters, which fortunately resolved with cessation of plasmapheresis. However, her renal failure did not improve, despite the undetectable anti-GBM antibody level, and is currently on HD.

## 4. Conclusions

Patients with advanced renal dysfunction with impaired excretory capacity undergoing plasma exchange with FFP anticoagulated with citrate require more frequent monitoring of acid base disturbance. These patients are prone to develop MA if subjected to high citrate load, which will metabolize to bicarbonate. Certain modifications in the plasmapheresis protocol such as albumin solution or cryoprecipitate with no citrate load can prevent the development of MA. The clinician should be able to identify this severe metabolic disorder, which if left untreated or unattended can lead to very sinister outcomes including the tissue hypoxia, neuromuscular excitability, and fatal arrhythmia.

## Figures and Tables

**Table 1:** Laboratory parameters (on admission day and later on after initiation of hemodialysis and plasmapheresis).

Parameters	On admission	Day 1	Day 2	Day 4	Day 5	Day 6	Day 7	Day 9	Day 12
pH	7.31	7.42	7.56	7.61	7.68	7.47	7.51	7.50	7.47
PCO_2_ (mmHg)	52	39	28	26.4	20.9	25.2	39.3	38	41.2
PO_2_ (mmHg)	40	129	320	162	129	99	198	216	192
Sodium (mEq/mL)	145	143	143	141	138	138	131	138	138
Potassium (mEq/mL)	4.4	4.3	2.9	3.8	4.4	4.4	3.9	2.9	2.8
Bicarbonate (mEq/mL)	21	26	34	27	28	17	23	29	25
Chloride (mEq/mL)	102	103	101	105	108	108	92	96	93
BUN (mg/dL)	29	49	13	35	17	17	56	24	41
Creatinine (mg/dL)	2.8	8.7	2.7	4.5	2.3	2.3	7.8	4.7	6.3
Hematocrit %	27.4	17.6	24.8	25.8	25	25	24.2	22	22.8
ANCA-MPO		>8				22.8			3.1
Anti-GBM antibody		7.4				2.8			1

## Abbreviations

ANCA-MPO: Antineutrophilic cytoplasmic antibodies myeloperoxidase
anti-GBM: Anti-glomerular basement membrane antibodies
MA: Metabolic alkalosis
GN: Glomerulonephritis
CT: Computerized tomography
FFP: Fresh frozen plasma.
